# Does the economic recession influence the incidence of pertussis in a cosmopolitan European city?

**DOI:** 10.1186/s12889-019-6448-3

**Published:** 2019-02-04

**Authors:** Sílvia Brugueras, Cristina Rius, Joan-Pau Millet, Martí Casals, Joan A. Caylà, Barcelona Pertussis Working Group, Pere Simón, Anna de Andrés, Roser Clos, Montse Ricart, Miriam Ros, Maria José Santomà, Pilar Gorrindo, Pilar Palau, Montse Cunillé, Eva Masdeu, Ingrid Avellanés, Jose Ángel Rodrigo, Gema Codina, Maria Teresa Martin, Juan José González, Sònia Brió, Jessica del Carpio, Andrea Otero, Álvaro Díaz, Núria López, Montse Vaqué, Pere Sala, Marta Calsina, Yolanda Meije, Eulàlia Guasch, Maria Rosa Pascual, Carme Palasi, Anna Luz Moro, Manel Enrubia, Gemma Monsó, Joan Martí Solé, Paloma Rodríguez, Helena Pallarès, Antoni Salvà, Ángeles Ferrández, Ana María Hostalot, María Carmen Muñoz, Anna de Andrés

**Affiliations:** 1 Servei d’epidemiologia, Agència de Salut Pública de Barcelona, Barcelona, Spain; 2 Centro de Investigación Biomédica en Red de Epidemiología y Salud Pública (CIBERESP), Barcelona, Spain; 3 Departamento de Pediatría, Obstetricia y Ginecología y Medicina Preventiva, Facultad de Medicina, Universidad Autónoma de Barcelona, Barcelona, Spain; 4 Tuberculosis Research Unit Foundation (fuiTB), Barcelona, Spain; 5 Sport and Physical Activity Studies Centre (CEEAF), University of Vic-Central University of Catalonia (UVic-UCC), Barcelona, Spain

**Keywords:** Bordetella pertussis, Cities, Economic recession, Epidemiology, Pertussis vaccine, Whooping cough

## Abstract

**Background:**

In the last few years, pertussis has re-emerged worldwide. The aim of this article is to study how the incidence of the disease has evolved in Barcelona city over a 16-year period, and determine which factors are associated with the evolution of the disease. We discuss the causes of the observed changes considering different possibilities such as vaccination coverage, vaccine effectiveness, increased surveillance or the effect of the current economic recession.

**Methods:**

We performed a cross-sectional, observational, population-based descriptive study using data for the 2000–2015 period from the notifiable diseases register maintained by Barcelona Public Health Agency. We used Poisson regression to compute adjusted odds ratios (aOR) and their corresponding 95% confidence intervals (CI).

**Results:**

A total of 1791 cases were registered. The incidence of the disease increased throughout the city from 2011 onwards. While children under 1 year of age had the highest-incidence and were the most at risk (aOR = 27.18, CI:23.51–31.44), we found that the age of affected children was higher in the last years. Incidence proportion (PRR) was lower among foreign-born children than native children (PRR = 0.43 CI:0.32–0.58). In the whole-cell vaccine period (2000–2004), the percentage of cases under 1 year of age who received the vaccine was lower than in 2005–2015 when the acellular vaccine was used (*p* = 0.01), suggesting a lower efficacy of the acellular vaccine. However, vaccination coverage in children under 6 years remained high (~ 90%), and there were no significant year-to-year variations (*p* = 0.757). Moreover, there did not appear to be any significant restrictions in medical care. According to the index of disposable household income (DHI), pertussis incidence increased from 2011 onwards in all neighbourhoods and remained higher in those with lower DHI.

**Conclusions:**

The noteworthy increase in pertussis incidence does not seem to be due to the economic recession, but to other factors here described.

## Background

Pertussis is an infectious respiratory disease caused by the Gram-negative *Bordetella pertussis* bacterium. Clinical symptoms include paroxysmal cough and inspiratory stridor, which may be followed by periods of apnea [1]. Although it is more common and is associated with more complication in children, it may occur in all age groups and there has been an increase in the age of detected cases of pertussis [1]. Adults suffer from less severe symptoms that are more difficult to diagnose, which results in increased risk of transmission within the family [2].

Pertussis is a Notifiable Disease (ND) that continues to be a public health issue worldwide despite broad vaccination coverage [3–5]. Globally, data from the European Centre for Disease Prevention and Control (ECDC) show an increase in the incidence of pertussis in the European Union (EU) from 2011 onwards. Several countries, including Spain, The Netherlands, Denmark, Belgium, Ireland and the United Kingdom (UK) witnessed an increase in incidence from 2011 onwards, while that in Italy and Greece has remain generally stable [1, 6, 7]. Non-European countries with broad vaccination coverage, such as the United States (US), Australia and Israel have also witnessed a re-emergence of pertussis. However, several reports indicate that this is not a global re-emergence of the disease [3, 7–9].

In several countries, such as the US, Mexico, Croatia and Thailand, economic recessions have been shown to have an impact on communicable diseases because of the subsequent increase in inequalities between different population groups. Disease transmission may increase because of increased contact, poorer living conditions, poorer access to treatment, and lower treatment adherence. In addition, public funding allocated to preventing such diseases may be affected by austerity policies, resulting in decreased care for individuals in greatest need [10, 11]. The current global economic recession started in 2008, and as yet there are no data on trends in the pertussis incidence in the context of this economic recession at local level.

In the current study we: (i) describe the clinical and epidemiological features of pertussis in Barcelona between 2000 and 2015; (ii) analyse how the incidence of the disease evolved; (iii) evaluate the effect of the type of vaccine administered among children under 1 year old (whole-cell vs acellular vaccine); (iiii) determine which factors are associated with the increasing incidence in the city between 2000 and 2015 considering variables such as the gender, the age group, the socioeconomic status and the period. Through a description of the disease over the years, we discuss the potential etiological factors of this increase and analyse whether the economic recession had an impact in pertussis incidence.

## Methods

### Design

Cross-sectional, observational, population-based descriptive study.

### Population

Pertussis cases of Barcelona notified by clinicians, microbiologists and public health nurses to the register of notifiable diseases (ND register) maintained by the Epidemiology Service of the Barcelona Public Health Agency (BPHA), Spain, between January 1st, 2000 and December 31st, 2015. The population of Barcelona during the period was between 1.503.884 inhabitants in 2000 and 1.609.550 in 2015 [12].

### Pertussis-related activities

Pertussis cases are reported to the BPHA, which carries out the follow-up of the cases and conducts the study of contacts. The study of contacts consists in reviewing the vaccination status of the contacts and adapting it to the immunisation schedule and administering prophylaxis to all contacts, following the pertussis guidelines of the from the health department of Catalonia. In order to know the vaccination status of the whole population there is a record in the Shared Medical History of Catalonia (HCCC) of the total population that receives healthcare in Catalonia. The immunization coverage in Barcelona has remained high. During the 2010–2015 period, primary vaccination among 1 year old children was between 90 and 95% and the complete vaccine coverage in young people aged 17 years was 68–77% (source: Sistema d’Informació dels Serveis d’Atenció Primària SISAP, Institut Català de la Salut). The immunisation program has remained the same during the study period except for the replacement of the whole-cell vaccine for the acellular vaccine. From 2004 to 2005 the acellular vaccine was administered at all doses.

### Case definition

Cases with a clinical suspicion of pertussis were those in which the typical clinical coughing symptoms were present for two weeks or longer, followed by one or several of the following symptoms: paroxysmal coughing, inspiratory stridor, vomit or apnea after coughing. No laboratory confirmation was available in such cases. A pertussis case was considered confirmed in patients with confirmed infection by *B. pertussis* using laboratory diagnostic tests, based on culture or Polymerase Chain Reaction (PCR). Confirmed cases also included patients who showed typical clinical symptoms and were epidemiologically related to any other case that had already been confirmed with laboratory tests. Both suspected cases and confirmed cases were considered pertussis cases for the purposes of the current study [13].

### Variables

Socio-demographic variables included gender, age (divided in 4 groups according to the most common distribution of pertussis: < 1, 1–14, 15–29 and > 30 years old), country of origin (native, foreign-born) and socio-economic status (divided in 5 groups, see below). Epidemiological variables were the following: conclusion (confirmed case, suspected case), association with other cases (sporadic, associated) and contact tracing. Clinical variables included symptoms (cough, paroxysmal coughing, inspiratory stridor, vomiting after coughing, apnoea, fever, pneumonia and convulsions), evolution (curation, death), vaccination, proper vaccination, laboratory diagnosis (culture, PCR) and hospital admission.

Vaccination variables included the number vaccinated cases (1 or more doses), non-vaccinated cases and cases of unknown vaccination status.

Proper vaccination status was defined as follows: people older than 2 years who had been given at least 4 vaccination doses; toddlers between 1 and 2 years of age who had been given at least 3 doses (proper primary vaccination), babies between 6 months and 1 year who had received at least 2 doses; babies between 3 and 6 months who had received at least 1 vaccination doses; all babies less than 3 months old [14]. The age groups used to analyse the vaccination variables were under 1 year old and under 6 years old.

To determine the socio-economic status of people suffering from pertussis, we classified neighbourhoods of residence -assigned according to the mailing address provided in the survey- according to the index of disposable household income (DHI). The DHI had the following categories: very high (> 159), high (126–159), average (100–125), average-low (between 79 and 99), and low (below 79). The DHI was constructed by analysing the following 5 indicators [15]:

1. Percentage of people with a degree: number of residents aged ≥25 years with a diploma, degree, or Ph.D. as a percentage of the total number of residents aged ≥25 years
2. Unemployment rate: number of unemployed people as a proportion of the total workforce
3. Private cars/1000 residents: number of cars owned by neighbourhood residents*1000/total residents
4. New high-power cars/total of new cars: number of cars less than two years old with more than 16 fiscal horsepower/the total number of cars under two years old (owned by residents; fiscal horsepower refers to a tax on high-powered vehicles in Spain)
5. Price of second-hand housing: Unit price of second-hand homes in euros.

Using the year variable, we created a new variable, period, to divide the years of the study into three periods: 2000–2004, 2005–2010 (after vaccines were changed from whole-cell vaccines to acellular vaccines) and 2011–2015 (years of the epidemic). We also created a new variable to identify the pre-recession (2000–2008) and recession periods (2009–2015).

To evaluate the effect of the type of vaccine administered, we created a vaccination period variable and classified children under 1 year old in either the whole-cell vaccine period (2000–2004) or the acellular vaccine period (2005–2015), depending on the type of vaccine received.

We also considered incidence differences between children under 15 years old foreign-born and natives.

### Information sources

Data were obtained from epidemiological surveys carried out by the Barcelona ND register. We used Barcelona city’s local census to obtain data on the total population in the city each year, distributed according to gender, age group, and neighbourhood. The DHI was constructed by Barcelona City Council Statistics Department [15].

### Statistical methods

We conducted a descriptive study of pertussis cases in Barcelona between 2000 and 2015, and calculated the annual incidence proportion of pertussis in the city overall, and according to sex, age group, DHI, and country of origin (number of cases per 100,000 residents). Incidence proportions were obtained by dividing the number of notified pertussis cases by the total population in each group. We also computed the incidence trend line that best fit the observed data. Incidence proportion rate ratio (PRR) in foreign-born under 15 years old versus natives was calculated.

As subanalysis, we compared vaccination coverage before and after the economic recession. We also determined the proportion of cases among children under 1 year old who had received either whole-cell or acellular vaccination, and used univariate analysis to evaluate potential significant differences.

To study the possible risk factors for the incidence of notified pertussis cases at the multivariate level, a generalized linear model (GLM) was used assuming the frequency of the cases followed a Poisson distribution. We performed this analysis using aggregated data (with incidence as the dependent variable) to determine the effect of the following variables on the incidence trend: gender, age group, DHI, and period. The model was adjusted for the variables gender, age group, DHI and period. As the offset of this model the logarithm of the number of population exposures in each group was used. We excluded 6 cases because they lacked data for the neighbourhood variable.

All analyses were performed using SPPS v18.0 and the statistical package R (The R Foundation for Statistical Computing, Vienna, Austria), version 3.1.1. Statistical significance was set at *p* < 0.05.

## Results

A total of 1791 cases of pertussis were included, of which 6.3% were diagnosed between 2000 and 2004, 16.0% between 2005 and 2010, and 77.8% between 2011 and 2015. There was 87.7% of cases classified as confirmed cases. Globally, 41% of cases were confirmed by diagnostic tests, 13.9% by epidemiological contact, and 45.2% using both methods. The remaining 12.3% of cases were classified as suspected cases. 55.9% of the cases were female, 84.9% were natives, and we observed an increase in the average age of cases during the study period. Most cases were from low-average and low DHI neighbourhoods. We also observed an increase in the detection of associated cases in the 2011–2015 period, as well as in the number of contact studies performed (Table 1).

**Table 1:** Distribution of pertussis cases according to epidemiological variables

	2000–2004 N (%)	2005–2010 N (%)	2011–2015 N (%)	Total N (%)
All	112 (6.3)	286 (16.0)	1393 (77.8)	1791 (100.0)
Confirmed	97 (86.6)	220 (76.9)	1253 (89.9)	1570 (87.7)
Suspected	15 (13.4)	66 (23.1)	140 (10.1)	221 (12.3)
Gender				
Female	69 (61.6)	160 (55.9)	772 (55.4)	1001 (55.9)
Male	43 (38.4)	124 (43.4)	621 (44.6)	788 (44.0)
Age group				
< 1 year	83 (74.1)	128 (45.1)	221 (15.9)	432 (24.1)
1–14 years	20 (17.9)	89 (31.3)	782 (56.1)	891 (49.7)
15–29 years	6 (5.4)	22 (7.7)	63 (4.5)	91 (5.1)
> 30 years	3 (2.7)	45 (15.8)	327 (23.5)	375 (20.9)
Country of origin				
Native	83 (74.1)	220 (76.9)	1217 (87.4)	1520 (84.9)
Foreign-born	7 (6.3)	26 (9.1)	122 (8.8)	155 (8.7)
Unknown	22 (19.6)	40 (14.0)	54 (3.9)	116 (6.5)
Index of Disposable Household Income (DHI)				
Very high	4 (3.6)	15 (5.2)	77 (5.5)	96 (5.4)
High	4 (3.6)	18 (6.3)	77 (5.5)	99 (5.5)
Average	19 (17.0)	58 (20.3)	325 (23.3)	402 (22.4)
Low-average	41 (36.6)	90 (31.5)	498 (35.8)	629 (35.1)
Low	41 (36.6)	105 (36.7)	413 (29.6)	559 (31.2)
Unknown	3 (2.7)	0 (0.0)	3 (0.2)	6 (0.3)
Case type				
Associated	18 (16.1)	127 (44.4)	824 (59.2)	969 (54.1)
Sporadic	94 (83.9)	159 (55.6)	569 (40.8)	822 (45.9)
Contact tracing				
Yes	36 (32.1)	216 (75.5)	1130 (81.1)	1382 (77.2)
No	76 (67.9)	70 (24.5)	263 (18.9)	409 (22.8)
Vaccination				
Yes	92 (82.1)	236 (82.5)	1005 (72.1)	1333 (74.4)
No	8 (7.1)	25 (8.7)	97 (7.0)	130 (7.3)
Unknown	12 (10.7)	25 (8.7)	291 (20.9)	328 (18.3)
Hospital admission in < 1 year (*N* = 432)				
Yes	64 (77.1)	83 (64.8)	105 (47.5)	252 (58.3)
No	19 (22.9)	45 (35.2)	116 (52.5)	180 (41.7)

Barcelona, 2000–2015

The following clinical symptoms were observed in cases: coughing that lasted longer than two weeks (77.8%), paroxysmal coughing (61.3%), inspiratory stridor (27.2%), post-coughing vomiting (29.5%), apnoea (9.5%), fever (5.9%), pneumonia (0.9%), and convulsions (0.1%). There were three deaths during the study period, one in 2008 and two in 2012.

In most cases, hospital admission involved children under 1 year (252/274, 92.0%), and there was a general decrease over time in the percentage of cases under 1 year of age who were admitted to hospital (Table 1).

Vaccination coverage remained high, although we found that the proportion of cases with unknown vaccination status increased during the 2011–2015 period, a period in which cases were also found to be generally older. More than 90% of children under 6 years (*N* = 791) were vaccinated, 88.9% were properly vaccinated according to their age, and 5.7% were non-vaccinated. Note that parents who are against vaccines only accounted for 3.9% of all cases in children under 6 years, and 68.9% of cases involving non-vaccinated children. Vaccination coverage before (91.3%) and during (90.6%) the economic recession remained very similar (*p* = 0.757). Analysing vaccination percentage according to DHI, we found that richer neighbourhoods (very high DHI) included a higher percentage of non-vaccinated cases, as well as a higher percentage of non-vaccinated children under 6 years and under 1 year (*p* < 0.05). Similarly, among vaccinated cases, the highest percentage of incorrect vaccination was observed in very high DHI neighbourhoods in children under 6 years, and in high DHI neighbourhoods in children under 1 year (Table 2).

**Table 2:** Vaccination status in pertussis cases according to age group and disposable household income. Barcelona, 2000–2015

	Age group	Vaccinated N (%)	Non-vaccinated N (%)	Unknown vaccination status N (%)	Proper vaccination in vaccinated cases N (%)
All categories (*N* = 1791)	Overall	1333 (74.4)	130 (7.3)	328 (18.3)	1146 (86.2)
	< 6 years	718 (90.8)	45 (5.7)	28 (3.5)	635 (88.9)
	< 1 year	406 (94.0)	15 (3.5)	11 (2.5)	371 (92.3)
Very high DHI (*N* = 96)	Overall	65 (67.7)	12 (12.5)	19 (19.8)	52 (80.0)
	< 6 years	37 (78.7)	8 (17.0)	2 (4.3)	31 (83.8)
	< 1 year	16 (76.2)	4 (19.1)	1 (4.8)	15 (93.8)
High DHI (*N* = 99)	Overall	73 (73.7)	11 (11.1)	15 (15.2)	60 (82.2)
	< 6 years	36 (85.7)	4 (9.5)	2 (4.8)	32 (88.9)
	< 1 year	18 (94.7)	0 (0.0)	1 (5.3)	15 (83.3)
Average DHI (*N* = 402)	Overall	280 (69.7)	29 (7.2)	93 (23.1)	239 (85.4)
	< 6 years	148 (88.6)	9 (5.4)	10 (6.0)	127 (85.8)
	< 1 year	95 (94.1)	3 (3.0)	3 (3.0)	87 (91.6)
Average-low DHI (*N* = 629)	Overall	464 (73.8)	42 (6.7)	123 (19.6)	401 (86.4)
	< 6 years	236 (91.1)	15 (5.8)	8 (3.1)	212 (89.8)
	< 1 year	120 (94.5)	5 (3.9)	2 (1.6)	109 (90.8)
Low DHI (*N* = 559)	Overall	447 (80.0)	36 (6.4)	76 (13.6)	394 (88.1)
	< 6 years	257 (95.2)	9 (3.3)	4 (1.5)	233 (90.7)
	< 1 year	153 (96.8)	3 (1.9)	2 (1.3)	145 (94.8)

*DHI* disposable household income

In the whole-cell vaccine period (2000–2004), 88.0% of cases under 1 year were vaccinated versus the 95.5% of cases under 1 year vaccinated in the acellular vaccine period (2005–2015) (*p* = 0.01). 82.2% of these were properly vaccinated according to their age between 2000 and 2004, and 94.6% in the 2005–2015 period (*p* < 0.001).

**Fig. 1:**
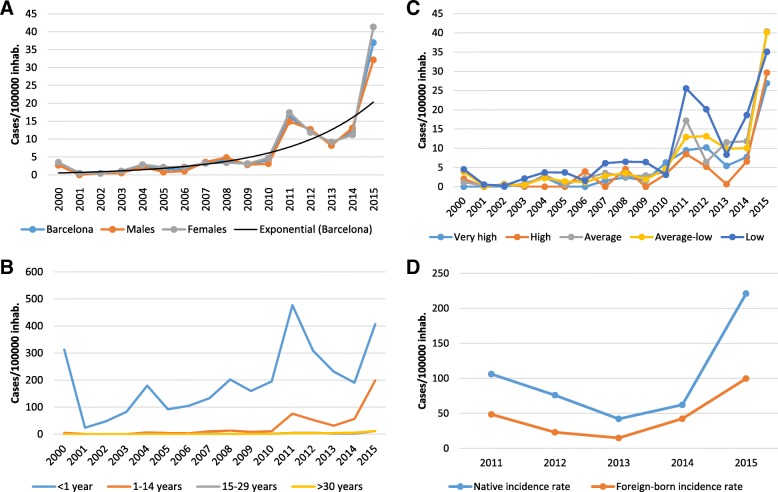
Incidence of pertussis in Barcelona, 2000–2015. **a** Incidence of pertussis in Barcelona according to sex, 2000–2015. **b** Incidence of pertussis in Barcelona according to age group, 2000–2015. **c** Incidence of pertussis in Barcelona according to index of Disposable Household Income, 2000–2015. **d** Incidence of pertussis among under 15-year-olds according to country of origin, Barcelona 2011–2015

We observed a sustained increase (R^2^ = 0.748 see exponential curve, Fig. 1a) in the incidence of pertussis in Barcelona 2011 onwards, from 3.1 cases/100,000 residents in 2000 to 16.2/100,000 in 2011, and, following a brief decline, a rise to 37.0 cases/100,000 in 2015. This trend was similar in both males and females (Fig. 1a).

In terms of age, the highest incidence observed during the study period was among children under 1 year old. The incidence remained very low in the rest of the population until 2011, after which we observed an increase among 1- to 14-year-olds that persisted until 2015 (Fig. 1b).

Grouping cases according to DHI, we did not observe a stable distribution during years of low incidence, although the incidence was generally higher in poorer neighbourhoods. From 2011 onwards, incidence increased in all neighbourhoods, and remained higher in those with lower DHI (Fig. 1c).

In terms of country of origin, we found that the incidence in children under 15 years old was higher among natives than among foreign-born (PRR = 0.43 CI: 0.32–0.58) (Fig. 1d).

Multivariate analysis showed that the increased incidence observed between 2000 and 2015 was associated with being female (aOR = 1.12, CI:1.02–1.23) and being a case during the 2011–2015 period (aOR = 1.75, CI:1.54–2.00). We also observed trend related to age-group, with the strongest association among children less than 1 year old (aOR = 27.18, CI:23.51–31.44). In terms of socio-economic status, we did not observe marked differences between neighbourhoods, although there was a general tendency (Table 3).

**Table 3:** Analysis of factors associated with pertussis incidence in Barcelona, 2000–2015

Variables	Categories	cOR	95%CI	aOR	95%CI
Gender	Male	1		1	
	Female	0.87	0.80–0.96	1.12	1.02–1.23
Age group	< 1 year	23.13	20.13–26.57	27.18	23.51–31.44
	1–14 years	6.64	5.89–7.50	6.80	6.03–7.69
	15–29 years	1.70	1.34–2.12	1.76	1.39–2.20
	> 30 years	1		1	
DHI	V.high-high	1		1	
	Average	1.21	1.02–1.44	1.31	1.10–1.55
	Average-low	1.63	1.39–1.91	1.64	1.40–1.94
	Low	1.71	1.46–2.02	1.62	1.37–1.91
Period	2000–2004	2.23	1.78–2.77	1.11	0.88–1.38
	2005–2010	1		1	
	2011–2015	1.24	1.09–1.41	1.75	1.54–2.00

Adjusted for the variables gender, age group, DHI and period

*DHI* disposable household income, *cOR* crude odds ratio, *aOR* adjusted odds ratio, *CI* confidence interval

## Discussion

In this study we have described evolution of pertussis in Barcelona city between 2000 and 2015. We observed a steady increase in incidence since 2010, which is in line with the trends observed elsewhere in Spain and in other European countries [1, 7]. The trend in pertussis incidence in recent decades follows a U-shaped curve, both in Spain and in other countries, such as the US and the UK. This is a similar pattern to that described for other communicable diseases, and requires greater focus on this disease and its control [6, 7, 9, 16]. Despite the periodicity of the disease we can see in other studies that the last epidemic wave of pertussis shows an increasing trend of the disease [6, 14, 17].

This increase does not seem to be related either to limitations in healthcare assistance (healthcare for communicable diseases was maintained in Barcelona, despite the economic recession and Royal Decree 16/12 which hinders access to the healthcare card among immigrants [18]) or to vaccination issues (vaccination coverage in Barcelona remained high over the years). The global rise in cases with unknown vaccination status was due to an increase in the age of cases; such a rise is not observed in the under 6 years and under 1 year age groups. Although the action of the anti-vaccination movement have been associated with a higher disease risk, this remains a minority group in Barcelona [19]. Several Spanish studies have shown the need for new vaccination strategies and more effective vaccines, given that a high percentage of cases is properly vaccinated [6, 14, 20]. Globally, several authors have suggested that vaccination strategies should be reformulated, as a result of their limited efficacy [5, 7, 21].

The percentage of cases under 1 year of age who received the vaccine was lower in 2000–2004 (when the whole-cell vaccine was used) than in 2005–2015 (in which the acellular vaccine was used). Moreover, the latter period was characterised by a higher proportion of properly vaccinated cases. These results agree with the hypothesis of lower efficacy of the acellular vaccine than the whole-cell vaccine [7, 22, 23].

Apart from the lower efficacy of the acellular vaccine, the increased incidence could be due to a genetic change in the bacteria. Bacterial antigens are known to change over time, and they no longer coincide with the antigenic profile of currently vaccines [24]. The increased incidence could also be due to faster, more sensitive diagnostic tests, improved epidemiological surveillance, better registry systems, and greater awareness by healthcare professionals [3, 7]. However, US- and UK-based study suggests that asymptomatic transmission could be key for the re-emergence of pertussis [25]. Moreover, pertussis often goes unrecognized among patients. The lack of characteristic symptoms is more common in adults, as opposed to children. In this sense, a review of studies from several countries showed the high seroprevalence of *B. pertussis* compared to the number of diseases that were notified [26]. This findings emphasise the need for better and maintained epidemiological surveillance in the entire population.

While children under 1 year are the most vulnerable age group, and the group with the highest disease incidence, we observed an increase among children older than 1 year and among teenagers. This increase in the age of cases may be due to greater awareness among healthcare professionals, leading to increased diagnosis among teenagers and adults (symptoms are less severe in these groups and thus harder to identify). The absence of characteristic symptoms results in greater diagnosis delay, such that other individuals are exposed to infected individuals for longer. This increase in age could also be due to reduced acquired immunity over time, which is attributed to the change in vaccine from whole-cell to an acellular vaccine and to reduced natural immunity after several years of low disease incidence, which does not allow immunity to be strengthened through naturally occurring disease [3, 6, 22, 23, 27, 28]. This decrease in immunity may have caused increased transmission between older children, teenagers and adults, who would in turn transmit the disease to younger children [2, 29, 30]. Other studies have also described increased reporting of cases at older ages, despite correct vaccination coverage [4, 9, 31–33]. This change highlights a need for a booster dose in vaccination schedules during teenage years.

The decline in the number of cases under 1 year of age who were admitted to hospital could be because formerly only the most severe cases were notified, because of improved hospital admission criteria, or because the disease appears in less severe forms that do not require hospitalization. This could also be related to earlier more precise diagnoses, and the use of faster, more sensitive diagnostic techniques.

Moreover, native children aged 0–14 years show higher disease incidence than among foreign-born children, in spite of the economic recession. This could be because low-income countries still use the whole-cell vaccine, which provides a longer-lasting immunity. In addition, immigrant children from countries with a high incidence of pertussis would have higher natural immunity.

Examining neighbourhoods according to DHI, we found that those with very high DHI had a lower incidence of pertussis. However, although these neighbourhoods have fewer cases, they show a higher percentage of non-vaccinated and not correctly vaccinated cases. Thus, some cases of infection may have occurred because of improper vaccination. As for all communicable disease, most cases of pertussis cluster in disadvantaged neighbourhoods in large cities, where poorer living conditions such as poor hygiene or overcrowding in small apartments leads to increased disease transmission [34–36]. However, note that health-related actions should not be limited to these neighbourhoods, as multivariate analysis shows that all neighbourhoods in the city are at risk of a similar increase in incidence. This shows that, despite the economic recession, neighbourhoods with low DHI have not been disproportionately affected.

Our multivariate analysis showed a remarkable association between the pertussis and children under 1 year of age. Thus, this age group should be the focus of resources and future health-related actions, and it would be also be necessary to promote vaccination in pregnant women.

Although economic recessions have previously been linked to negative impacts on child health [37], the current recession does not seem to have affected access to the healthcare system or vaccination coverage, and foreign-born children were not more seriously afflicted than native children. Thus, the increase detected is due to other factors mentioned above, such as vaccine effectiveness, reduced immunity, gene changes in *B. pertussis*, and the availability of new diagnostic tests.

A possible limitation of the study is under-diagnosis and under-notification of the disease, mostly in adult population, as well as the absence of data on their vaccination status. Nonetheless, this is a population-based study of Barcelona city that includes 16 years of data from the BPHA registry. In addition, socio-economic level was determined using a DHI indicator created by Barcelona City Council. Given the high number of cases and their follow-up, we consider this to be a valid study of considerable scientific interest.

## Conclusions

The noteworthy increase in pertussis incidence does not seem to be due to the economic recession, but to other factors such as vaccine effectivity or genetic change in the bacteria. The increased incidence could also be due to faster, more sensitive diagnostic tests, improved epidemiological surveillance, better registry systems and greater awareness by healthcare professionals. Another key factor could be asymptomatic transmission and the absence of characteristic symptoms among infected people. Although we observed an increase in the age of cases, children under 1 year continue to be the most vulnerable age group, and the group with the highest disease incidence. According to the index of DHI, we observed that neighbourhoods with low DHI have not been disproportionately affected. However, pertussis incidence increased from 2011 onwards in all neighbourhoods and remained higher in those with lower DHI.

In conclusion, we consider that the following measures should be undertaken to improve the epidemiology of the disease: (i) prioritize preventive measures to prevent transmission to most vulnerable groups including children under 1 year such as vaccinate pregnant women; (ii) intensify epidemiological surveillance in the entire population.

## Abbreviations

aOR: adjusted odds ratio
BPHA: Barcelona Public Health Agency
CI: confidence intervals
cOR: crude odds ratio
DHI: disposable household income
ECDC: European Centre for Disease Prevention and Control
EU: European Union
GLM: generalized linear model
ND: Notifiable Disease
OR: odds ratio
PCR: Polymerase Chain Reaction
PRR: Incidence proportion rate ratio
UK: United Kingdom
US: United States
